# Antiscience, Vaccine Hesitancy, and Pandemic Responses: Highlights from the Asia Pacific Summit on Infectious Diseases and Immunization †

**DOI:** 10.3390/vaccines12121336

**Published:** 2024-11-27

**Authors:** Ananta Seth, Nick Sevdalis, Zulkifli Ismail, Sri Rezeki Hadinegoro, Heidi J. Larson, Tikki Pangestu

**Affiliations:** 1 Asia-Pacific Immunization Coalition, Yong Loo Lin School of Medicine, National University of Singapore, Singapore 119228, Singapore; heidi.larson@lshtm.ac.uk (H.J.L.);; 2 Yong Loo Lin School of Medicine, National University of Singapore, Singapore 119228, Singapore; 3 Centre for Behavioural and Implementation Science Interventions, Yong Loo Lin School of Medicine, National University of Singapore, Singapore 117597, Singapore; 4 Department of Pediatrics, KPJ Selangor Specialist Hospital, Shah Alam 40300, Malaysia; 5 Asia Dengue Voice and Action Group, Hong Kong SAR, China; 6 Department of Child Health, Faculty of Medicine, Universitas Indonesia, Jakarta 10430, Indonesia; 7 London School of Hygiene & Tropical Medicine, London WC1E 7HT, UK; 8 Institute for Health Metrics & Evaluation, University of Washington, Seattle, WA 98195, USA

**Keywords:** antiscience, vaccines, infectious diseases, immunization, public health, pandemic response, immunization programs, vaccine efficacy, vaccine access, vaccine hesitancy, antivaccine activism

## Abstract

The recent resurgence of mpox highlights the urgent need for rethinking vaccination strategies globally, underscored by the painful memories of past public health crises where delayed responses and inequitable vaccine distribution exacerbated the spread of infectious diseases. The inaugural APIC-ADVA Asia Pacific Summit on Infectious Diseases and Immunization, themed “Vaccination for All: Access, Confidence and Equity (ACE)”, was held in Singapore from 31 October to 1 November 2023 in an attempt to present best practices and hard-won insights from battling COVID-19 and other pandemics in the Asia-Pacific region. This summit was co-convened by the Asia-Pacific Immunization Coalition (APIC) and Asia Dengue Voice and Action (ADVA). Local, regional, and international experts from academia, research and representatives from the Ministries of Health, the World Health Organization (WHO), and the International Vaccine Institute (IVI) participated in the 2 day summit. With more than 230 speakers and delegates from over 15 countries, and 4 symposia over 2 full days, the first APIC-ADVA Asia Pacific Summit on Infectious Diseases and Immunization highlighted critical issues affecting vaccine access, confidence, and equity, and emphasized the importance of safeguarding the world from existing infections and future pandemics through immunization.

## 1. Introduction

Public health crises (such as the recent resurgence of mpox) where delayed responses and inequitable vaccine access intensified the spread of infectious diseases, have highlighted the pressing need to rethink global vaccination strategies [1]. The first APIC-ADVA Asia Pacific Summit on Infectious Diseases and Immunization, co-convened by the Asia-Pacific Immunization Coalition (APIC) and Asia Dengue Voice and Action (ADVA), was held in Singapore from 31 October to 1 November 2023. The summit, themed “Vaccination for All: Access, Confidence and Equity (ACE)”, included local, regional, and international experts including those from the Ministries of Health of several Asian countries, the International Vaccine Institute (IVI), and global and regional representatives from the World Health Organization (WHO).

The summit emphasized the importance of life-course immunization as an important strategy for preventing infectious diseases and promoting public health. Although it involves vaccinating individuals across their lifespan, from infancy through adulthood and into old age, many people in the Asia-Pacific region do not have access to life course immunization, particularly in low- and middle-income countries. Antivaccine activism and vaccine hesitancy are also issues that must be addressed. A major concern for national governments and international health agencies, including the WHO, is how antivaccine activism might now spill over into other vaccination programs [2]. This might have a role in promoting resistance to the introduction of new malaria or other vaccines intended specifically for the world’s low- and middle-income countries (LMICs) [3]. A globalizing antivaccine movement could also extend to all routine childhood immunizations and hasten the re-emergence of measles, pertussis, or even polio, globally [4].

Resolute multi-sectoral partnerships, building advocacy and engagement with the public, and tackling pressing challenges of vaccine inequity and vaccine hesitancy are key to attaining equitable access to vaccines across the Asia Pacific region.

Key highlights from the first APIC-ADVA Asia Pacific Summit on Infectious Diseases and Immunization are presented here.

## 2. Experiences from the SEA Region: Providing Access, Building Confidence and Ensuring Equity in Vaccines

Immunization programs play a critical role in safeguarding public health across the region. These initiatives aim to reduce the incidence of vaccine-preventable diseases, such as measles, polio, hepatitis, malaria, dengue, influenza and COVID-19. By targeting diverse populations, from infants to the elderly, these programs ensure widespread immunity and disease prevention. Collaborative efforts between governments, international organizations, and healthcare providers are crucial for the success of these initiatives. They include vaccination campaigns, education, and outreach efforts to address vaccine hesitancy. Continuous monitoring and adapting to emerging health threats are essential components, ensuring the region remains resilient against infectious diseases and achieves improved health outcomes.

The Dengue-Zero Project is a public–private partnership in Thailand driven by a memorandum of understanding (MOU) detailing how national government entities, academic institutions, and private sectors can collaborate to achieve three goals by 2026: a reduction in dengue mortality by 25% annually, a reduction in dengue mortality rate to less than 0.01%, and a reduction in breeding sites of Aedes mosquitoes in the community to less than 5 per 100 households.

In Indonesia, dengue cases can be found in almost all cities and districts. In general, the incidence of dengue infection is high in urban areas with dense populations. The country faced a significant outbreak in 2016 and despite existing vector control strategies, the dengue burden in Indonesia remains high, with increasing impact over the years. The country aims to reduce dengue incidence to less than 10 per 100,000 in at least 90% of districts and cities by 2025, the case fatality rate to 0.5% in 2025, and zero dengue deaths by 2030 [5]. The Ministry of Health’s six-pillar National Strategy with Innovative Dengue Prevention includes innovative technologies like Wolbachia and preventive vaccines.

Indonesia has introduced two dengue vaccines, CYD-TDV (approved in 2016) and TAK-003 (2022). Dengue vaccine development has been challenging because of the need to provide protection against all four dengue serotypes [6,7]. TAK-003 has, in the phase 3 TIDES 4.5-year-long trial, demonstrated efficacy in preventing symptomatic cases (61% efficacy) and hospitalizations (84% efficacy), and was generally well tolerated without any important safety risks identified in both seropositive and seronegative patients [8].

Malaysia’s National Immunization Program (NIP) covers 13 vaccine-preventable diseases, and the newest addition is the pneumococcal vaccine introduced in 2020. Challenges to vaccine coverage were faced during the COVID-19 pandemic, including reduced health facility visits. Despite the challenges, Malaysia managed to trace defaults and restore coverage to over 95%. Emerging challenges to coverage comprise vaccine refusal and hesitancy, suboptimal coverage in certain regions, and issues with non-citizen populations. Refusal reasons include concerns about vaccine contents, Halal-Haram issues, and fears about artificial intelligence. To address these challenges, surveillance of vaccine refusal cases has been ongoing since 2013 alongside the development of a manual for counseling resistant parents.

To deal with vaccine hesitancy, Malaysia introduced its “Immunise4life” program, which focuses on community education and nationwide training of healthcare providers to effectively communicate with hesitant parents. To ensure vaccine equity and access, Malaysia employs outreach activities, personalized care in health clinics, efficient vaccine distribution, and strong political will to ensure a continuous operational budget for vaccines and service delivery. Vaccine access inequities are also addressed, for example, by providing free polio vaccines for non-citizens.

Future plans include a nationwide, non-selective measles–rubella vaccination campaign, the introduction of the Tdap vaccine for pregnant mothers, and the establishment of an immunization registry. Malaysia also aims to enhance advocacy, collaborate with partners, and utilize integrated monitoring systems to strengthen its immunization program.

## 3. Addressing Vaccine Hesitancy by Facilitating Evidence Use in National Immunization Programs

Facilitating evidence use in national immunization programs is critical to countering the rise of antiscience, vaccine hesitancy and antivaccine activism, ensuring that public health policies are informed by science and bolstering public confidence in vaccination efforts. By integrating scientific research and data-driven insights, policymakers can design and implement more effective vaccination campaigns. This involves collecting and analyzing epidemiological data, monitoring vaccine efficacy, and understanding population health trends. Collaborative efforts with global health organizations, researchers, and local stakeholders ensure that immunization policies are based on the latest evidence. Continuous education and training for healthcare providers also enhance program effectiveness. Ultimately, evidence-based decision-making improves vaccine coverage, addresses public concerns, and adapts to emerging health threats, ensuring robust and responsive national immunization programs.

The World Health Organization’s Immunization Agenda 2030 mission is to ensure that everyone, at every age, receives the full benefits of vaccination, extending beyond health to overall well-being [9]. The WHO recognizes differences in vaccine development priorities between high- and low-income countries. The multifaceted stakeholders involved in the vaccine development pipeline are categorized into primary demanders, agents, countries, and enablers. A unified strategy is necessary to guide these stakeholders toward a common goal, minimizing unnecessary delays in product development and accelerating vaccine uptake.

The “Full Value of Vaccine Assessments (FVVA)” concept has the potential to help spur vaccine development [10]. The concept comprises three key functions: assessment, decision-making, and communication. This framework aims to standardize value assessments, engage end users transparently, and improve coordination among stakeholders, and it is used by the WHO to guide vaccine developers about the need for vaccines that address the specific burdens of low- and middle-income countries (the introduction gap) and the importance of considering factors like strain coverage, duration of protection, storage conditions, and price tolerance [11].

In Indonesia, several challenges remain in achieving >95% vaccine coverage, including pockets of unvaccinated children who have not been reached by its National Child Immunization Program and inequality of immunization services (only 47.1% districts reached 95% of MCV2 coverage). Other challenges include low compliance by health workers in the implementation of an national electronic immunization registry, limited number of immunization and surveillance staff and high staff turnover, insufficient regular training/capacity building, stock outages of reagents for confirming outbreaks, poor surveillance coverage (19% of Indonesia’s districts are considered “silent” in terms of case reporting), delays in responding to outbreaks in hard-to-reach areas due to lack of accessibility and resources, and vaccination hesitancy.

Vaccination hesitancy is a community obstacle in Indonesia, fueled by concerns and fears, which affect routine immunization and acceptance of multiple injections. Survey results indicate a 64.02% acceptance rate for immunization, with 22.6% refusal. Understanding vaccine benefits was identified as crucial, along with perceived service quality and the demand for information on safe immunization practices.

Indonesia has chosen to align its immunization strategy with the World Health Organization’s Western Pacific Region strategic framework for Vaccine Preventable Diseases (VPDs) and Immunization, focusing on managing health intelligence on VPDs and immunization, strengthening and expanding systems and programs, and enhancing preparedness and response to public health emergencies [12]. It has committed to improving health worker compliance with the electronic registry, conducting cascade training to improve the capacity of health workers—both for vaccination and surveillance and strengthening micro planning—and strengthening local government commitment. It is also working on securing laboratory supplies, ensuring vaccine safety, and garnering support for vaccination coverage at the local government level.

In Thailand, the process of introducing a vaccine for approval is aligned with the WHO’s FVVA framework, integrating disease burden, surveillance, and societal impact criteria. Criteria for new vaccines include disease burden, surveillance quality, societal impact, and the presence of safe and efficacious vaccines. The decision-making process involves analyzing vaccine effectiveness, potential competition, and conducting cost-benefit, cost-effectiveness, and cost-saving analyses. Program feasibility, logistics, public acceptance, and political awareness are key considerations.

The decision-making structure involves subcommittees, including the Subcommittee on the Advisory Committee on Immunization Program (ACIP), which communicates directly with the national vaccine committee chaired by the prime minister. The information is then presented to the National List of Essential Vaccines Subcommittee, integrating health economics evaluations conducted by the Health Intervention and Technology Assessment Program (HITAP).

If the vaccine is deemed cost-effective, negotiations on price are initiated. If approved, the Health Insurance Fund under the Universal Health Coverage (UHC) program funds vaccine coverage. Thailand’s UHC program, implemented since 2002, ensures all vaccines in the national program are provided free of charge. The final clearance involves presenting information to the Royal Thai Government and undergoing the budgetary process. The entire mechanism aims to harmonize decisions and acceptance levels, making the policy on vaccine introduction evidence-based and data-driven.

## 4. Upgrading Vaccine Policy Making for the Modern World

Vaccine policymaking for the modern world involves embracing innovative technologies, data-driven approaches, and global collaboration. Modern policies must integrate real-time surveillance data, advanced research, and genomic insights to swiftly respond to emerging threats.

Data-driven insights include evidence that Dengvaxia, the first dengue vaccine to be approved, has a paradoxical efficacy across different age groups. While the vaccine appeared protective for those above 9 years old, a relative risk of 7.45 for hospitalization in the youngest age group raised concerns [13]. Further analysis revealed a potential serostatus effect, indicating harm for seronegative individuals [14]. The WHO responded by recommending restricted use in high seroprevalence settings and advising serological testing before vaccination [15].

Unlike Dengvaxia, TAK-003 exhibits protective efficacy for all, eliminating the need for complex eligibility criteria. Trial results for TAK-003 have revealed positive impacts across diverse serological statuses and age groups [16,17]. The economic evaluation of TAK-003 involves assessing the reduction in the cost of treating dengue illness against the additional cost of vaccination. The potential decay of vaccine efficacy over time is included, with a focus on ongoing monitoring to ensure continued effectiveness. Economic projections for TAK-003 in highly endemic regions like Brazil showcase potential reductions in dengue cases, indicating population-level benefits [18]. However, challenges in cost-effectiveness considerations, with the threshold cost per fully vaccinated person being lower than anticipated, require competitive pricing.

## 5. Inequities in Global Vaccine Access

Global vaccine inequities persist due to issues with coordination, financing, delivery, and the rise of antiscience and anti-vaccine advocacy, which undermines vaccine access, public confidence, and immunization efforts. These challenges not only hinder vaccine efficacy and pandemic response but also have significant impacts on economic recovery, public health, and the success of immunization programs worldwide. COVID-19 global vaccination coverage remains unequal; despite progress, low-income countries still face challenges, with vaccine coverage at 28.4% compared to the WHO’s target of 40% of each eligible country by the end of 2021 and 70% by mid-2022 [19]. Equitable access is not only the right thing to do, but is also linked to uneven recovery: countries with higher rates of vaccine coverage recover faster from the economic disruption caused by COVID-19, with low vaccination rates contributing to a drag on recovery elsewhere [20]. While output has surpassed pre-pandemic levels in most of the larger East Asia and Pacific economies, recovery has been uneven across the region. Long-term consequences of the pandemic include slower long-term growth leading to increasing inequality in the learning progress of children, issues concerning women, and losses incurred by micro, small and medium enterprises [21].

To address equitable vaccine distribution, a range of strategies has been implemented across various global organizations.

COVAX aimed to ensure vaccine equity, but it faced challenges like financing delays, reliance on a single supplier, export restrictions, and slow initial responses from international organizations [22]. The complex landscape of vaccine nationalism and global supply chain issues significantly affected COVAX’s effectiveness during the COVID-19 pandemic. A coordinated response was needed from day zero, but slow response times and inadequate collaboration until mid-2021 among international agencies, governments, and organizations, as well as financing complications, including issues with COVAX’s fundraising and the World Bank’s eligibility criteria, further complicated the situation.

Potential solutions to these challenges include revisiting financing models, exploring blended finance options, and fostering collaboration among different stakeholders. There is a need for flexible, transparent, and coordinated approaches, particularly in the context of the proposed pandemic fund. The World Bank’s proposed pandemic fund, with its focus on disease surveillance, lab systems, and health workforce strengthening, signifies a step towards addressing future pandemics more effectively [23].

The International Vaccine Institute (IVI) is an international research organization which supports vaccine R and D for low- and middle-income countries [24]. By diversifying funding and collaborating with organizations like Wellcome Trust, IVI conducts annual training on vaccinology and runs a global training hub on biomanufacturing, supported by the Korean government and the WHO Academy. IVI’s initiatives extend to training programs for young scientists, collaborations on global health security, and supporting companies through technology transfer. IVI has completed 10 tech transfers, including products like oral cholera, typhoid conjugate, and dengue vaccines, showcasing its capabilities at different stages of vaccine development. IVI extends its reach globally, supporting pivotal studies in Nepal, training programs in Bangladesh, vaccination campaigns in Fiji, and it is committed to supporting Africa’s vaccine R and D ecosystem, demonstrating a commitment to equitable vaccine access. Efforts include tech transfer, strengthening regulatory environments, and providing training to achieve Africa’s ambitious vaccine production goals. It aims to contribute to tackling vaccine equity comprehensively by leveraging its projects in discovery, development, field studies, tech transfers, and training initiatives globally.

PATH is an international NGO with a rich history spanning over 40 years that is actively engaged in improving health equity through innovative approaches and partnerships [25]. The organization’s global footprint extends to more than 70 countries, showcasing its commitment to addressing health challenges on a broad scale. Its primary focus lies in enhancing immunization systems to ensure equitable access to vaccines. In Laos and Vietnam, PATH quadrupled vaccination rates by establishing committees and coordination in vaccination campaigns and routine immunization efforts for an effective response to changing situations; training staff on various immunization aspects, including cold chain supply, microplanning, data quality, and direct communication skills, to ensure a skilled workforce; providing continuous training through e-learning platforms and job aids to address resource shortages and turnover; generating vaccination lists through data review and developing microplanning digital tools to optimize resources and minimize wastage; utilizing mobile campaigns in remote and difficult sites to reach hard-to-reach populations, training locals and integrating direct communication; providing training on data quality, utilizing electronic registries (NIIS, DHIS2), and developing additional reporting mechanisms for better decision-making; and organizing diverse platforms for sharing lessons, such as panel discussions, review meetings, and dissemination workshops, to foster collaboration and replication of successful strategies.

These efforts collectively illustrate a global commitment to tackling vaccine equity through improved funding structures, innovative research collaborations, technology transfer, and local capacity building.

## 6. Improving Access to Immunization Amongst Older Adults in Asia Pacific

The Asia-Pacific region has an urgent need to enhance immunization access for older adults. A study on strategies to enhance this access was conducted, prompted by the region’s aging population and the need for preventive measures against infectious diseases, highlighted by the recent COVID-19 pandemic [26]. The study’s objectives were understanding barriers, identifying policy gaps, and exploring socio-cultural factors affecting immunization among older adults. Four diverse health systems—Australia, Singapore, Indonesia, and the Philippines—were chosen for in-depth analysis.

The study used a two-pronged approach involving a rapid literature review and key informant interviews. The findings were categorized into four key areas: complex inter-individual drivers of vaccination decision-making; community and systems-level drivers; persistent disparities across subpopulations; and lessons learned from past interventions. The individual drivers included perceived disease risk, awareness of vaccination, and perceptions of vaccine efficacy versus risks of harm. Community and systems-level factors encompassed access, family and friends’ influence, trust in peer groups, and the role of healthcare systems.

Key challenges identified included issues of access, cost, linguistic barriers, and the influence of family dynamics. Family members played a dual role, acting as both enablers and discouragers of vaccination. Trust in peer groups and the healthcare system, coupled with the government’s role in disseminating information, emerged as crucial influencers. Recommendations were made at three levels: individual, community, and systems. At the individual level, addressing knowledge gaps, persuading older adults about the value of vaccination, and enabling them through behavioral science interventions were suggested strategies. Community-level recommendations included fostering interconnectivity among stakeholders and leveraging community networks. The study proposed developing a national adult immunization program, reviewing guidelines and funding, and integrating technologies to enhance effectiveness.

## 7. Closing Remarks

Inequalities in access to vaccines, vaccine hesitancy and inequities in vaccine distribution continue to be challenges for the Asia-Pacific region. Continued and better integration of multi-sectoral collaboration (health, policy, education, environment), international mobilization of resources (research, funding, technology), continued and new financial sources and political commitment, strengthening of public health capacity, sustained community outreach efforts, innovations in vaccine technologies, and vaccine advocacy can help address these challenges. With a shift of focus to vaccines in the post-COVID-19 era, continuous collective efforts, and newer tools being developed, the world is coming closer to a goal of vaccination for all. The key topics addressed in this paper, including potential strategies and innovations, will continue to be addressed and further explored in the second APIC-ADVA Asia Pacific Summit on Infectious Diseases and Immunization, to be held on 11–13 February 2025 [27].
